# Perioperative fluid accumulation and clinical outcomes after cardiac surgery: a systematic review and Bayesian meta-analysis

**DOI:** 10.1186/s44158-026-00346-2

**Published:** 2026-01-23

**Authors:** Rafael Hortêncio Melo, Anelise Poluboiarinov Cappellaro, Victor Gomez Galeano, Amanda Pascoal Valle Felicio, Adrian Wong, Rogerio da Hora Passos

**Affiliations:** 1 Hospital Municipal Gilson de Cássia Marques de Carvalho, Hospital Israelita Albert Einstein, Av. Santa Catarina, 2785 - Vila Santa Catarina, São Paulo - SP, 04378-500 Brazil; 2 Centro Universitário Maurício de Nassau de Barreiras, BR 135 Km 01 2341, Rua Boa Sorte, Barreiras, BA 47800-970 Brazil; 3 Hospital Auxilio Mutuo San Pablo, Calle Santa Cruz, Urb # 70, Bayamón, 00956 Puerto Rico; 4 King’s College Hospital NHS Foundation Trust, The Guthrie Wing, Denmark Hill, London, SE5 9RS UK; 5 Hospital Israelita Albert Einstein, Av. Albert Einstein, 627/701 - Morumbi, São Paulo - SP, 05652-900 Brazil

**Keywords:** Fluid overload, Cardiac surgery, Perioperative care

## Abstract

**Background:**

Fluid accumulation is common in critically ill patients and has been associated with adverse outcomes. However, its impact on postoperative outcomes in cardiac surgery remains unclear.

**Purpose:**

To assess the association between perioperative fluid accumulation and clinical outcomes in adults undergoing cardiac surgery.

**Methods:**

We conducted a systematic review and meta-analysis of observational studies and randomized controlled trials. PubMed, Embase, and the Cochrane Library were searched through February 2025. Eligible studies enrolled adults (≥ 18 years) undergoing cardiac surgery and compared liberal versus restrictive fluid strategies or fluid-positive versus fluid-restrictive states. Outcomes included all-cause mortality, acute kidney injury (AKI), hospital and intensive care unit (ICU) length of stay, duration of mechanical ventilation, ICU readmission, and postoperative atrial fibrillation (POAF). Certainty of evidence was assessed using the GRADE framework.

**Results:**

Eighteen studies (15,052 patients) were included. In pooled analyses, fluid accumulation was associated with increased mortality (OR 1.65; 95% CI 1.03–2.63; *p *= 0.04), and fluid restriction was associated with decreased hospital stay (MD −1.02 days; 95% CI −1.67 to −0.37; *p* = 0.002). Bayesian analysis supported these findings, showing a 98.8% probability that restrictive fluid strategies reduce mortality and a 98.6% probability of shorter hospital stay. For AKI, the Bayesian model showed an 84.7% probability of benefit despite non-significant frequentist results. No significant associations were found for POAF, ICU stay, mechanical ventilation duration, or ICU readmissions. The certainty of evidence was low for randomized evidence and very low for observational data when assessed with GRADE.

**Conclusions:**

Perioperative fluid accumulation may be associated with worse postoperative outcomes, but the certainty of evidence is limited by heterogeneity and methodological variability across studies. These findings should be interpreted as hypothesis-generating and underscore the need for high-quality randomized trials to clarify safe fluid exposure thresholds and the role of individualized perioperative fluid management.

**Supplementary Information:**

The online version contains supplementary material available at 10.1186/s44158-026-00346-2.

## Key-points

- Fluid accumulation is associated with worse outcomes after cardiac surgery,
- Bayesian analysis confirmed a high probability of benefit of fluid restriction.
- These findings support individualized perioperative fluid management and highlight the need for high-quality trials.

**Supplementary Information:**

The online version contains supplementary material available at 10.1186/s44158-026-00346-2.

## Introduction

Fluid accumulation in critically ill patients is a persistent clinical concern and continues to be an active area of investigation. Recent trials investigating more restrictive or individualized fluid approaches have further challenged longstanding practices and highlighted the need for refined resuscitation protocols, underscoring the association between positive fluid balance and adverse outcomes [1, 2]. Patients undergoing cardiac surgery frequently require perioperative hemodynamic support, and fluid therapy is often used as a first-line strategy to maintain circulatory stability [3–5]. However, in the absence of protocolized resuscitation, this approach poses a significant risk of fluid overload [6], potentially leading to worse outcomes such as acute kidney injury (AKI), prolonged hospital length of stay (H-LOS), and increased mortality [7, 8]. Perioperative fluid exposure can be described either in terms of protocol-based strategies (e.g., goal-directed or fluid-sparing regimens) or as measured states of accumulation (e.g., positive fluid balance, weight gain, cumulative overload); these are distinct but related expressions of the same underlying exposure. Evidence regarding how these different patterns of fluid exposure affect postoperative outcomes in cardiac surgery remains limited, so we conducted a systematic review and meta-analysis to evaluate the association between fluid accumulation and clinical outcomes in adult patients undergoing cardiac surgery.

## Methods

This review was registered with the International Prospective Register of Systematic Reviews (PROSPERO) under registration number CRD420251033118 and was conducted in accordance with the Cochrane Collaboration guidelines and Preferred Reporting Items for Systematic Reviews and Meta-Analyses (PRISMA) statement [9, 10]. PRISMA checklists are presented in the Supplementary Material. This study involved secondary data from previously published studies, exempting it from institutional review board approval.

### Eligibility criteria

We included observational and randomized controlled trials that evaluated adult patients (aged ≥ 18 years) undergoing cardiac surgery. Eligible studies were those investigating the association between fluid accumulation and postoperative outcomes. We defined fluid accumulation as any comparison in which a group with a positive fluid balance or liberal fluid strategy was evaluated against a more restrictive or conservative strategy, with respect to the outcomes of interest.

The outcomes of interest included all-cause mortality, AKI, intensive care unit length of stay (ICU–LOS), H–LOS, duration of mechanical ventilation, readmissions to the intensive care unit (ICU), and incidence of postoperative atrial fibrillation (POAF). Studies were excluded if they met any of the following criteria: (1) pediatric population; (2) absence of a comparison group; (3) no reported outcomes of interest; and (4) conference abstracts, case reports, review articles, or editorials. Only English-language articles were included.

### Search strategy and data extraction

Two authors (A.C. and L.A.) systematically searched PubMed, Embase, and the Cochrane Library up to November 2025 using the following search terms: “cardiac surgery,” “fluid therapy,” and “fluid balance.” Details of the complete search strategy are provided in the Supplementary Material. Two authors (A.C. and L.A.) independently screened the literature, with any discrepancies resolved by a third author (R.M.). Additionally, a backward search (snowballing) and a forward search (citation-tracking) were conducted for the included articles and relevant literature reviews. Rayyan.ai [11] software was used to screen, select, and exclude duplicate studies.

### Definitions of fluid accumulation

Definitions of fluid accumulation varied across trials. To improve interpretability, we classified the exposure into a priori conceptual categories based on timing and metric: (1) intraoperative fluid overload, restricted to the surgical and/or cardiopulmonary bypass (CPB) period; (2) early postoperative, typically 12–48 h after surgery in the ICU or recovery unit; (3) cumulative or weight-based postoperative overload, defined over a longer postoperative window (e.g., ≥ 5–10% weight gain or cumulative fluid accumulation during the index hospitalization); and (4) protocol-based fluid strategies, in which patients were randomized to different fluid management protocols (e.g., conservative or COP-guided strategies versus usual care) without a prespecified numerical threshold for fluid overload. Protocol-based strategies and measured accumulation states are conceptually distinct but were treated as related exposure categories.

### Quality assessment

Two reviewers independently assessed the risk of bias for each study using the Cochrane Risk of Bias tool for randomized trials (RoB 2) for *randomized* studies according to the following domains: random sequence generation, allocation concealment, blinding of participants and personnel, blinding of outcome assessment, incomplete outcome data, selective outcome reporting, and other biases which categorize studies as having a low risk of bias, some concerns, or a high risk of bias [12]. For observational studies, the Risk of Bias In *Non-randomized* Studies of Interventions (ROBINS-I) tool was applied [13].

### Statistical analysis

Statistical analyses were performed using frequentist and Bayesian methods. For dichotomous outcomes, odds ratios (ORs) with corresponding 95% confidence intervals (CIs) were calculated, and *p*-values less than 0.05 were deemed significant for treatment effects. Continuous outcomes were summarized as mean differences (MDs) or standardised mean differences (SMDs), depending on the measurement scale. When studies reported medians with interquartile ranges or ranges instead of means and standard deviations, we estimated means and SDs using established methods for this purpose [14, 15]. Cochran’s Q test and I^2^ statistics were used to assess for heterogeneity; *p-values less than 0.10* and *I*^2^ > 75% were considered significant for heterogeneity. We used the DerSimonian and Laird random-effects model to account for heterogeneity among the studies. We planned to perform leave-one-out sensitivity analyses for all outcomes with statistically significant results.

Exploratory subgroup and sensitivity analyses were planned a priori to investigate sources of heterogeneity. First, we stratified trials according to the predefined exposure categories (intraoperative positive balance, early postoperative accumulation, cumulative or weight-based postoperative overload, and protocol-based fluid strategies) and repeated the meta-analysis when at least three studies contributed to a given category. Second, we conducted sensitivity analyses excluding protocol-based strategy trials from the primary models for the main outcomes, as well as leave-one-out analyses, to assess the robustness of the pooled estimates.

In addition to the primary frequentist analyses, we performed Bayesian random-effects meta-analyses in R (version 4.3.2) using the bayesmeta package [16]. For dichotomous outcomes, study effects were analyzed on the log-odds ratio scale; for continuous outcomes, mean differences (or standardized mean differences when required) were analyzed assuming normal sampling distributions around study-specific true effects, with true effects distributed as ϑi∼n(μ,τ^2^), where μ is the pooled effect and τ the between-study heterogeneity. We used vague priors for μ (normal (0,5) for mean differences; normal (0, 2.7) for log odds ratios). To minimize over-interpretation in the presence of heterogeneity, our primary heterogeneity prior for mortality was an empirically derived informative prior based on Turner et al. [17], whereas for all other outcomes we used a weakly informative prior for τ (half-normal (0.52) as recommended by Röver et al. [18]). Robustness was assessed by repeating analyses under wider heterogeneity priors, including a vague half-normal (1.52), and we report posterior point estimates with 95% credible intervals, posterior probabilities for pre-specified clinically relevant thresholds, and 95% posterior predictive intervals to reflect the range of effects plausibly expected in a future study.

### Certainty of evidence (GRADE)

A certainty of the evidence for the main outcomes was assessed using the GRADE (Grading of Recommendations Assessment, Development and Evaluation) framework [19, 20]. Given that our review included both randomized controlled trials and observational studies, bodies of evidence were evaluated separately by study design. Randomized evidence was initially rated as high certainty and downgraded when concerns were identified; observational evidence was initially rated as low certainty and could be downgraded or, when appropriate, upgraded.

## Results

**Fig. 1:**
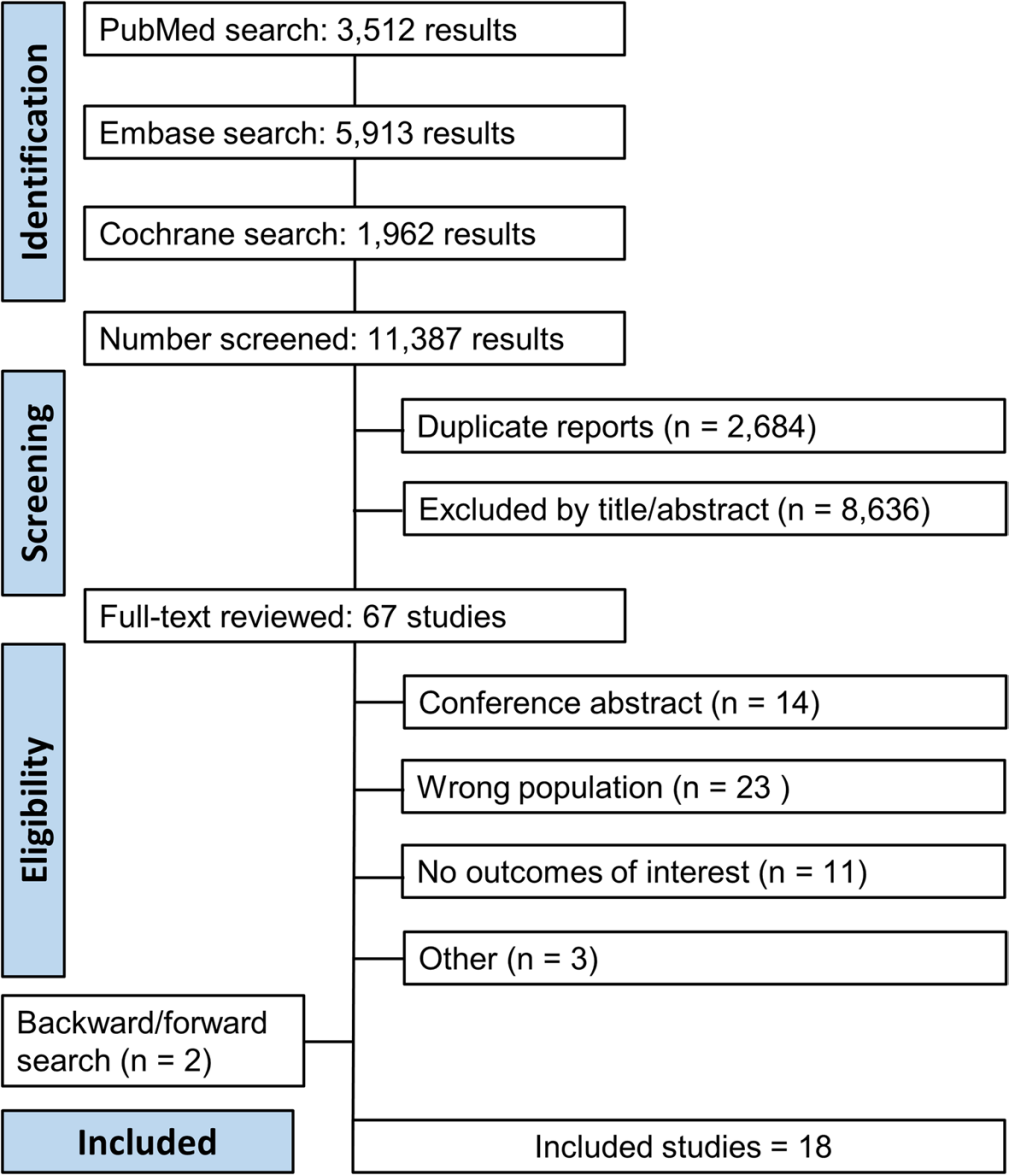
PRISMA flow diagram of study screening and selection

As shown in Fig. 1, the initial search identified 11,387 articles across the selected databases. After removing duplicates and screening titles and abstracts, 37 articles were considered potentially eligible and were retrieved for full-text review. Following the application of predefined inclusion criteria, 16 studies were selected for final inclusion. Two additional studies were identified through backward and forward citation tracking, resulting in a total of 18 included studies comprising 15,052 patients.

The baseline characteristics of the included studies are presented in Table 1. Four studies were *randomized* controlled trials (RCTs) [6, 24, 29, 31], six studies employed a prospective design, and the remaining studies were retrospective. The mean age varied from 56 to 70.2 years, and male prevalence ranged from 56.3% to 83.3%. Coronary artery bypass grafting (CABG) and valve surgery were the most common surgical interventions. The mean CBP time ranged from 63.3 to 175 min and was not reported in all studies.

**Table 1:** Baseline characteristics of included studies

Author, year	Study design	Patients number	Male *n*, (%)	Age, years (mean)	EuroSCORE (median)	HFrEF, *N* (%)	Surgery type
Chen (2021) [21]	Retrospective cohort	1522	857 (56.3)	56.3 ± 13.2	NA	NA	- Valve only 51.2%, CABG only 16.5%, CABG + valve 7.3% thoracic aorta 11.8%, other 13.2%
Shahidi Delshad (2020) [22]	Retrospective cohort	130	88 (67.7)	61.6	NA	N/A	CABG only
Haase-Fielitz (2017) [23]	Prospective cohort	282	197 (69.8)	68.6 ± 10.0	5.3 + 2.7	47 (16.7)	- Valve only 38.3%, CABG only 42.9%,CABG + valve 12.1%, other 7.5%
Fitzgerald (2015) [24]	Randomized	30	25 (83.3)	59.6 (12.1)	3.1	2 (0.06)	CABG only
Kambhampati (2012) [25]	Prospective cohort	100	60 (60)	61.4 ± 1.4	NA	10 (10)	- Valve only 40%, CABG only 32%,thoracic aorta 28%
Koc (2020) [26]	Retrospective cohort	373	262 (70.2)	68	5	94 (25.2)	- Valve only 19.5%, CABG only 55.5%, CABG + valve 12.2%, other 12.8%
Koskinen (2023) [27]	Prospective cohort	939	719 (76.5)	65.7 (10.5)	NA	114 (12.1)	- Valve only 47.6%, CABG only 46.4%, other 6%
Li (2018) [28]	Retrospective cohort	567	369 (65)	59	NA	NA	NA
Özdemir (2023) [29]	Randomized	60	46 (76.6)	62.2	3.2	NA	- Valve only 21.7%, CABG only 76.6%, CABG + valve 1.6%
Palomba (2022) [30]	Prospective cohort	586	384 (65.5)	62.6 (10.8)	NA	55 (9.4)	NA
Parke (2015) [31]	Randomized	144	117 (81.2)	65.4	2.3	NA	- Valve only 30.5%, CABG only 57.6%, CABG + valve 11.1%, other 0.8%
Parke (2021) [6]	Randomized	715	526 (73.5)	70	1.8	NA	- Valve only 24.4%, CABG only 43.4%, CABG + valve 20%, other 12.2%
Pradeep (2010) [32]	Retrospective cohort	1357	1074 (79.1)	65.4	NA	155 (11.4)	CABG 65.5%;
Shen (2018) [33]	Retrospective cohort	3899	2688 (68.9)	67.5 ± 22.7	NA	N/A	N/A
Smith (2020) [34]	Retrospective cohort	2327	1425 (61.2)	70.2	NA	NA	Aortic valve replacement
Stein (2012) [7]	Prospective cohort	502	308 (61.4)	62.4 ± 13.3	2.9	100 (19.9)	CABG only 51%; valve 36.3%; others 12.7%
Toraman (2004) [35]	Prospective cohort	1280	1011 (79)	59.8	3.1	448 (35)	CABG only
Xiao (2024) [36]	Retrospective cohort	239	195 (81.5)	62	NA	NA	CABG only 73.2%; CABG + valve 26.8%

*CABG* coronary artery by-pass grafting, *HFrEF* heart failure with reduced ejection fraction, *NA* not avaliable

Among RCTs, a restrictive strategy was defined as fluid infusion guided by hemodynamic monitoring parameters. Definitions of fluid overload varied across the observational studies, encompassing criteria such as percentage of weight gain, cumulative fluid balance within the first 12 to 24 h, and the total volume of fluid accumulation expressed in mL per kilogram of body weight. Study-level definitions of fluid overload and the corresponding fluid exposure categories are summarized in Table 2.

**Table 2:** Study-level definitions of fluid overload and classification by fluid exposure category

**Fluid exposure categories**	
**Intraoperative fluid balance**	**Fluid overload definition**
Palomba (2022) [30]	Intraoperative fluid balance > 2000 mL
Pradeep (2010) [32]	Intraoperative fluid balance > 3900 ml
Toraman (2004) [35]	Intraoperative fluid balance ≥ 500 ml
Xiao (2024) [36]	Fluid balance > 500 ml at the end of surgery
**Early postoperative fluid balance**	**Fluid overload definition**
Shahidi Delshad (2020) [22]	Fluid balance > 3000 ml in 24 h
Haase-Fielitz (2017) [23]	Fluid balance > 4000 ml in 24 h
Kambhampati (2012) [25]	Fluid balance > 2500 ml in 24 h
Koc (2020) [26]	Fluid balance > 3500 ml in 12 h
Li (2018) [28]	Positive daily fluid balance on day 2
Shen (2018) [33]	Fluid balance > 30 ml/kg in 48 h
Smith (2020) [34]	Fluid balance > 40 ml/kg in 24 h
**Cumulative/weight-based overload**	**Fluid overload definition**
Chen (2021) [21]	≥ 5% of fluid accumulation in 24 h (Total quantity of fluid received (L)—total amount of fluid eliminated (L)]/preoperative weight (kg) × 100)
Koskinen (2023) [27]	5% weight gain during index hospitalization
Stein (2012) [7]	≥ 10% of fluid accumulation during ICU stay (total quantity of fluid received (L)—total amount of fluid eliminated (L)]/preoperative weight (kg) × 100)
**Protocol-based fluid strategies**	**Fluid overload definition**
Fitzgerald (2015) [24]	Colloid osmotic pressure guided therapy and fluid balance in 24 h
Özdemir (2023) [29]	NA
Parke (2015) [31]	NA
Parke (2021) [6]	NA

### Outcomes

#### All-cause mortality

##### All-cause mortality

**Fig. 2:**
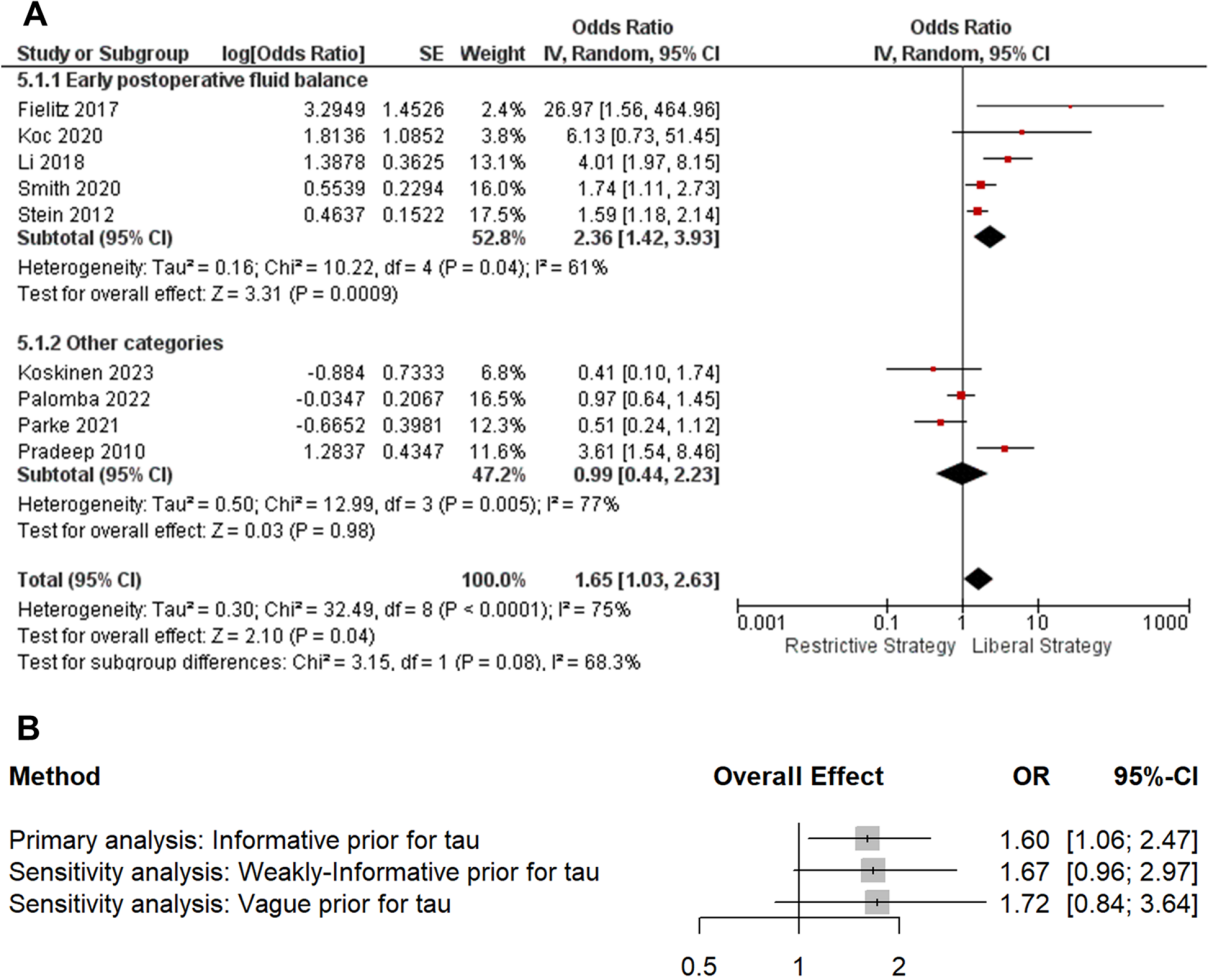
Mortality—frequentist subgroup analysis and Bayesian sensitivity to heterogeneity priors. **A** Random-effects subgroup meta-analysis (early postoperative fluid balance vs other categories) comparing liberal versus restrictive fluid strategies. **B** Bayesian random-effects pooled effects under informative, weakly informative, and vague priors for between-study heterogeneity (τ). Odds ratios are liberal vs restrictive; OR > 1 indicates higher mortality with liberal strategies

In the frequentist analysis, a statistically significant increase in mortality was observed in patients managed with liberal versus restrictive fluid strategies (OR 1.65; 95% CI 1.03–2.63; *p* = 0.04; *I*^2^ = 75%) (Fig. 2). In exploratory subgroup analyses according to the predefined exposure categories of fluid accumulation, the direction of effect was generally consistent across subgroups, although confidence intervals were wide and heterogeneity persisted (Fig. 2).

Bayesian random-effects meta-analysis using an empirically derived informative prior for between-study heterogeneity suggested that restrictive fluid management was associated with lower mortality than liberal strategies. The posterior pooled odds ratio for liberal versus restrictive management was 1.60 (95% CrI 1.06–2.47), corresponding to a 98.8% posterior probability that liberal strategies increase mortality (OR > 1). The posterior probability that liberal management confers a clinically meaningful mortality reduction relative to restriction (OR ≤ 0.8) was 0.2%, supporting restrictive management under this prior. However, heterogeneity remained substantial (τ = 0.39, 95% CrI 0.04–0.80), and the 95% posterior predictive interval was wide (OR 0.57–4.70), indicating that the effect in a future study could plausibly range from benefit to marked harm (predictive probability that OR > 1: 86.3%). Sensitivity analyses using weaker and vague priors for τ yielded similar point estimates but wider credible intervals crossing 1 (weakly informative: OR 1.67, 95% CrI 0.96–2.97; vague: OR 1.72, 95% CrI 0.84–3.64), while remaining directionally consistent and closely aligned with the frequentist estimate (Figure S1).

#### Hospital length of stay

**Fig. 3:**
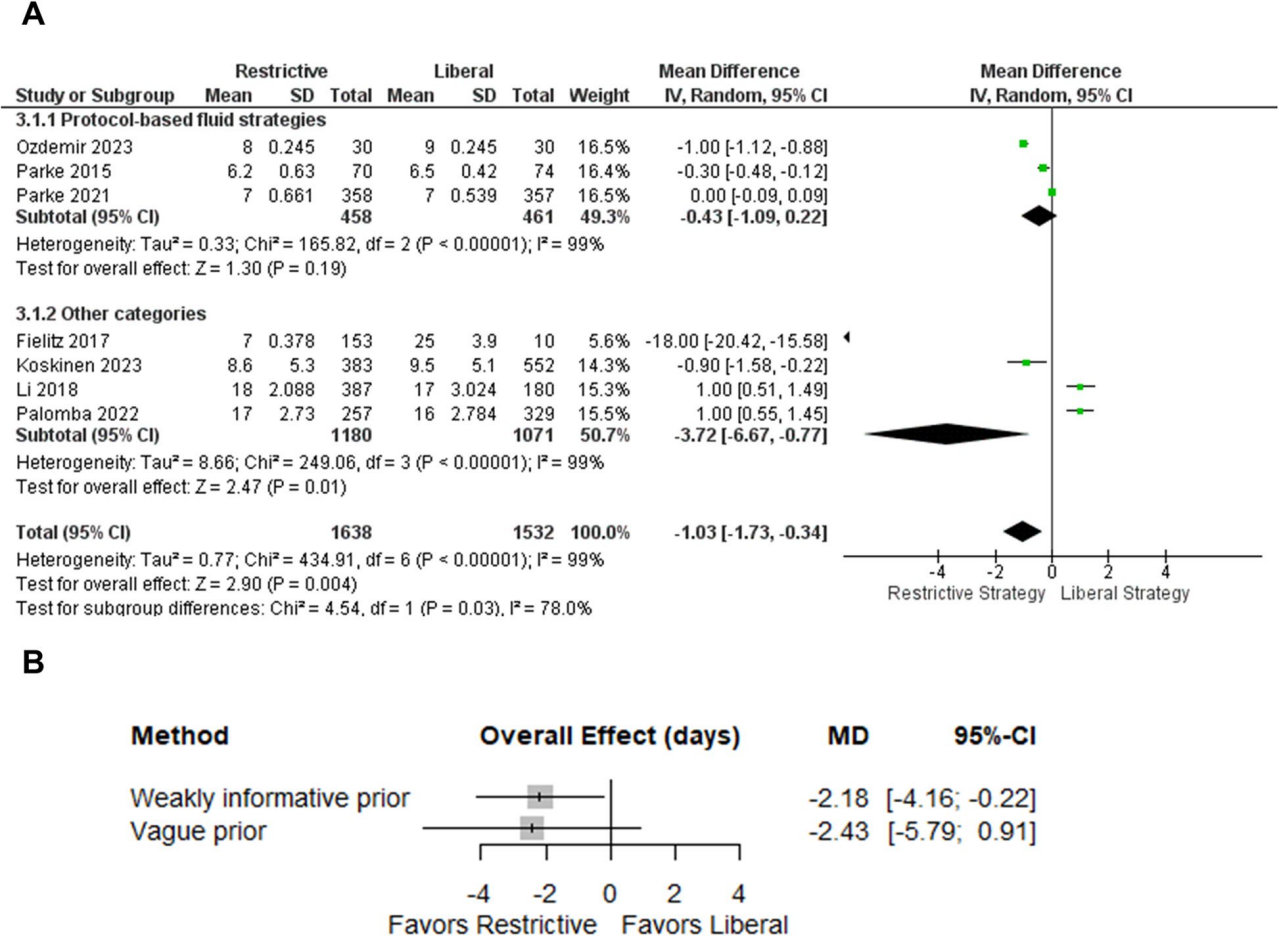
Hospital length of stay—frequentist subgroup analysis and Bayesian sensitivity to heterogeneity priors. **A** Frequentist random-effects meta-analysis comparing restrictive versus liberal fluid strategies, stratified by subgroup (protocol-based fluid strategies vs. other categories). **B** Bayesian random-effects sensitivity analysis showing posterior pooled mean differences and 95% credible intervals under weakly informative and vague priors for between-study heterogeneity (τ). Mean differences are expressed as restrictive minus liberal; MD < 0 indicates shorter length of stay with restrictive strategies

In the frequentist analysis, a restrictive strategy was associated with a statistically significant reduction in H-LOS compared with a liberal strategy (MD −1.02 days; 95% CI −1.67 to −0.37; *p* = 0.002; *I*^2^ = 98%)—Fig. 3. Subgroup analyses by exposure category suggested more pronounced effects in observational studies than in randomized trials: in the subgroup of protocol-based RCTs, the pooled effect on H-LOS was not statistically significant (MD −0.43 days; 95% CI −1.09 to 0.22), whereas observational studies showed a larger, but imprecise, reduction in length of stay (MD −3.07 days; 95% CI −5.43 to −0.71). However, substantial heterogeneity persisted in all subgroups, limiting firm conclusions regarding any specific category or design as a source of variability.

Bayesian random-effects meta-analysis (weakly informative τ prior) suggested that restrictive fluid management reduced hospital length of stay compared with liberal strategies (MD −2.18 days, 95% CrI −4.16 to −0.22). This corresponded to a 98.6% posterior probability of any reduction (Pr[MD < 0]), an 88.4% probability of at least a 1-day reduction, and a 57.1% probability of at least a 2-day reduction. However, heterogeneity was very high (*I*^2^ = 99%) and the posterior predictive interval was wide (−7.68 to 3.30), indicating that a future study could plausibly show little or no benefit (predictive Pr[MD ≤ 0] = 78.6%). Under a vague τ prior, the point estimate was similar but less precise and crossed zero (MD −2.43, 95% CrI −5.79 to 0.91) (Figure S2).

#### Acute kidney injury

**Fig. 4:**
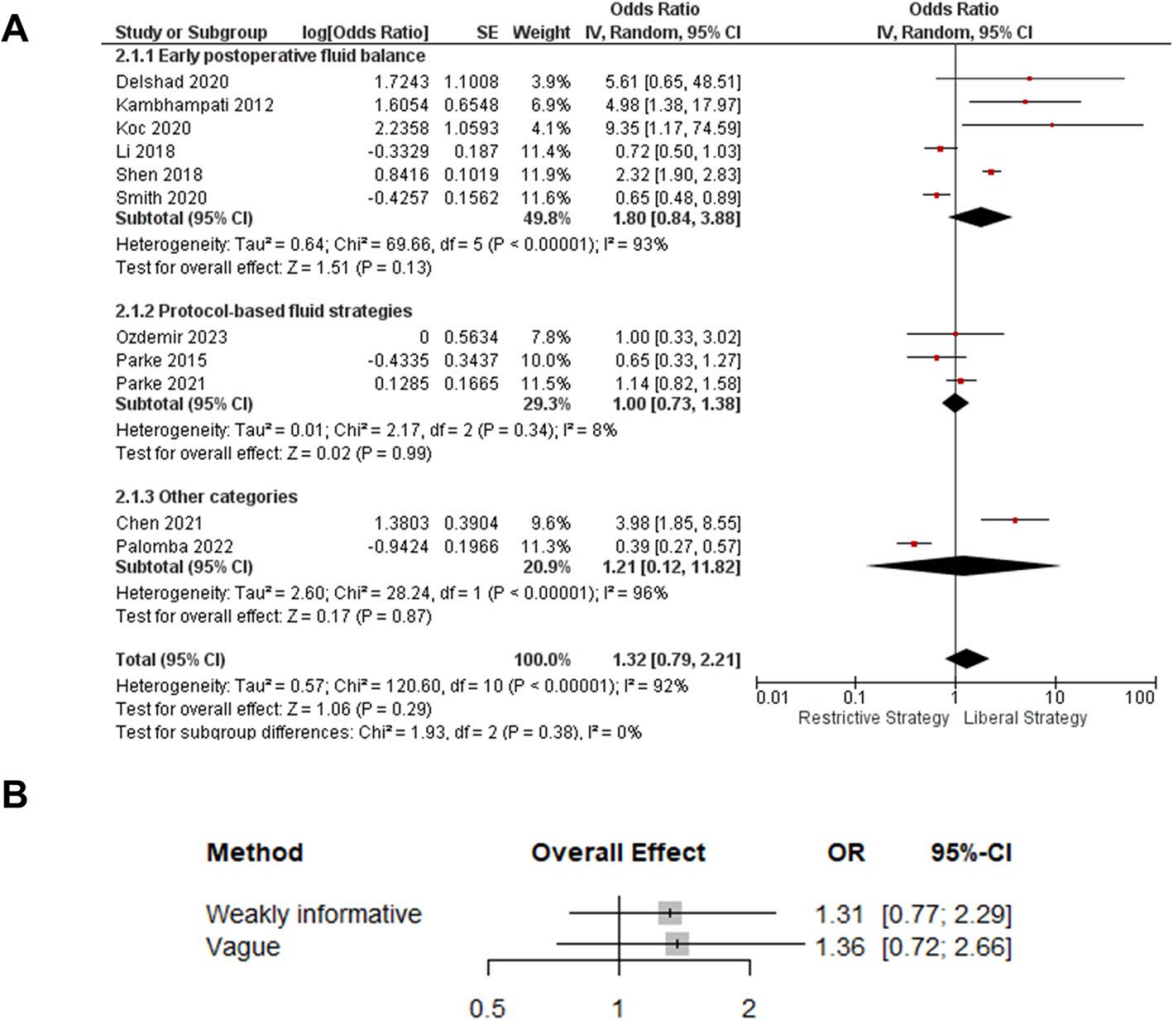
Acute kidney injury—frequentist subgroup analysis and Bayesian sensitivity to heterogeneity priors. **A** Frequentist random-effects meta-analysis comparing liberal versus restrictive fluid strategies, stratified by subgroup (early postoperative fluid balance, protocol-based fluid strategies, and other categories). **B** Bayesian random-effects sensitivity analysis showing posterior pooled odds ratios and 95% credible intervals under weakly informative and vague priors for between-study heterogeneity (τ). Odds ratios are expressed as liberal vs. restrictive; OR > 1 indicates higher AKI risk with liberal strategies

In the frequentist random-effects meta-analysis, the overall effect was not statistically significant (OR 1.32, 95% CI 0.79–2.21; *I*^2^ = 92%)—Fig. 4. Subgroup estimates were broadly directionally consistent but imprecise, and the test for subgroup differences was not significant (*p* = 0.38).

Bayesian random-effects meta-analysis (weakly informative τ prior) yielded a pooled odds ratio (liberal vs. restrictive) of 1.31 (95% CrI 0.77–2.29). This corresponded to an 84.7% posterior probability that liberal strategies increase AKI risk (OR > 1) and a 3.0% probability of a clinically meaningful reduction with liberal management (OR ≤ 0.8). However, heterogeneity was substantial (τ = 0.74, 95% CrI 0.42–1.15; frequentist *I*^2^ = 92%), and the posterior predictive interval was wide (OR 0.25–7.13), indicating considerable uncertainty for a future study (predictive Pr[OR ≤ 1] = 36.8%). Under a vague τ prior, the point estimate was similar but less precise and crossed the null (OR 1.36, 95% CrI 0.72–2.66) (Figure S3).

#### Postoperative atrial fibrillation

In the frequentist analysis, no statistically significant difference was observed between the strategies (OR 1.47; 95% CI, 0.82 to 2.63; *p* = 0.19; *I*^2^ = 43%) (Figure S4).

Bayesian random-effects meta-analysis (weakly informative heterogeneity prior) suggested higher atrial fibrillation with a liberal versus restrictive fluid strategy (posterior OR 1.43, 95% CrI 0.78–2.96), corresponding to an 89.8% posterior probability that the liberal strategy increases atrial fibrillation (OR > 1) (Figure S3). The probability that liberal management provides a clinically meaningful reduction (OR ≤ 0.8) was low (2.2%). Using a more vague heterogeneity prior yielded a similar point estimate but greater imprecision (OR 1.55, 95% CrI 0.62–4.74).

#### ICU length of stay

In the frequentist analysis, no statistically significant difference was observed between strategies (MD −0.05 days; 95% CI, −0.15 to 0.06; *p* = 0.37; *I*^2^ = 35%) (Figure S5).

In a Bayesian random-effects meta-analysis, restrictive versus liberal fluid management showed essentially no difference in ICU length of stay (posterior MD −0.02 days, 95% CrI −0.09 to 0.04). The probability that the restrictive strategy reduces ICU LOS was 78.9%, but the probability of a clinically meaningful ≥ 1-day reduction was negligible (P[MD ≤ −1] = 2.3%) (Figure S5). Between-study heterogeneity was low (τ = 0.05, 95% CrI 0.00–0.13), and the 95% posterior predictive interval was narrow (−0.19 to 0.14 days). The results were unchanged under a vague heterogeneity prior (MD −0.02 days, 95% CrI −0.09 to 0.04; τ = 0.05, 95% CrI 0.00–0.14), indicating evidence of no clinically meaningful effect on ICU LOS.

### Duration of mechanical ventilation

In the frequentist analysis, there was no statistically significant difference in mechanical ventilation duration between strategies (MD −0.60 days; 95% CI, −1.50 to 0.31; *p* = 0.19; *I*^2^ = 95%) (Figure S6).

Bayesian random-effects meta-analysis showed a small, uncertain reduction in mechanical ventilation duration with a restrictive strategy. Under a weakly informative heterogeneity prior, the posterior mean difference was −0.64 h (95% CrI −1.56 to 0.40), with an 89.3% posterior probability that restrictive strategies reduce ventilation duration (MD < 0) and a 20.9% probability of a reduction ≥ 1 h (MD ≤ −1) (Figure S6). Between-study heterogeneity was very high (τ = 0.91, 95% CrI 0.45–1.45), and the posterior predictive distribution suggested a 78.5% probability that a future trial would favor restriction (MD ≤ 0). Using a vague heterogeneity prior yielded a similar but less precise estimate (MD −0.51 h, 95% CrI −1.96 to 1.09; τ = 1.39, 95% CrI 0.55–2.63).

### Readmissions to the ICU

The frequentist analysis found no statistically significant difference between both strategies and fluid management (OR 0.87; 95% CI, 0.45 to 1.67; *p* = 0.67; *I*^2^ = 66%) (Figure S7).

Under a Bayesian random-effects model (weakly informative heterogeneity prior), the pooled OR for liberal versus restrictive fluid management was 0.90 (95% CrI 0.44–1.82), corresponding to a 66.0% posterior probability that the liberal strategy reduces readmissions (OR < 1) and a 34.0% probability that restrictive strategies reduce readmissions (OR > 1) (Figure S7). Between-study heterogeneity was moderate-to-high (τ = 0.43, 95% CrI 0.00–0.99), and the posterior predictive distribution suggested a 60.4% probability that a future trial would favor the liberal strategy (OR < 1), although with substantial uncertainty. A sensitivity analysis using a vague heterogeneity prior yielded a similar point estimate but wider uncertainty (OR 0.94, 95% CrI 0.30–3.23; τ = 0.75, 95% CrI 0.00–2.03).

### Quality assessment

**Fig. 5:**
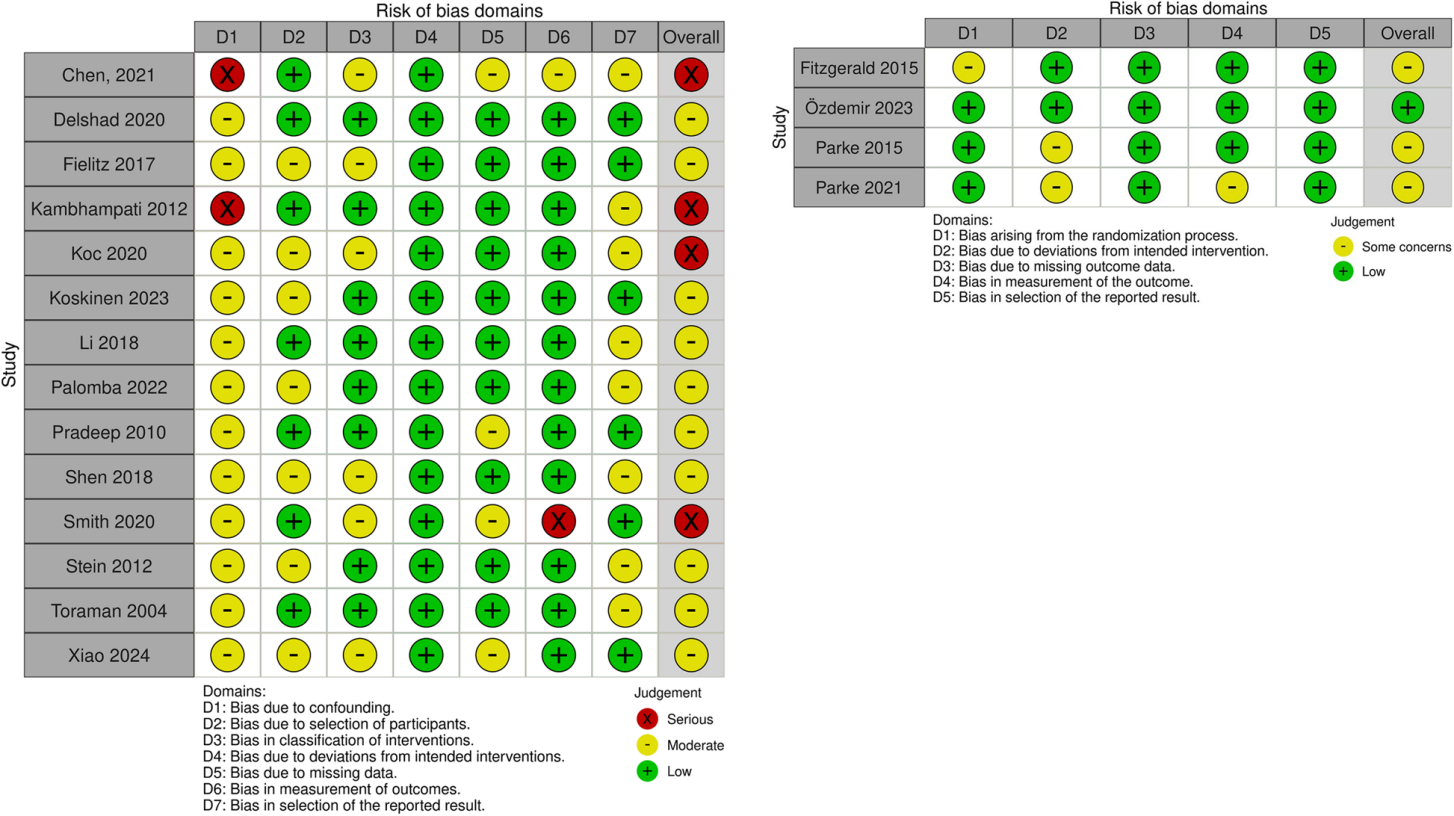
Quality assessment of included studies, ROBINS-I(left) for observational studies and ROB-2 (right) for randomized studies

Quality assessment of the observational studies was conducted using the ROBINS-I tool (Fig. 5). Overall, most studies were considered to have a moderate risk of bias, predominantly owing to confounding (domain D1) and selection of the reported result (domain D7). Three studies were rated as having a serious risk of bias owing to concerns in multiple domains, including confounding and participant selection.

For randomized controlled trials, the RoB 2 tool was applied (Fig. 5). Two studies were rated as having an overall low risk of bias, while two were judged to have some concerns, primarily related to the randomization process (D1) and deviations from the intended interventions (D2). No study was classified as having a high risk of bias.

### Publication bias

Visual inspection of the funnel plot revealed slight asymmetry (Figure S8); however, Egger’s regression test (*p* = 0.50) did not provide evidence of small-study effect.

### Sensitivity analysis

Subgroup results for mortality, H-LOS, and AKI are presented alongside the primary analyses and were generally consistent with the overall effects but remained imprecise and highly heterogeneous (Figs. 2, 3, 4). Subgroup analyses for the other outcomes showed a similar pattern and are detailed in the Supplementary Material.

The leave-one-out sensitivity analysis for mortality (Figure S9) demonstrated that the overall effect size remained relatively stable, with ORs ranging from 1.42 (95% CI) 0.90–2.25) to 1.91 (95% CI 1.21–3.00) across iterations. The pooled OR varied minimally.

When each study was sequentially omitted, indicating that no single study had a disproportionate impact on the overall estimate. Heterogeneity remained substantial throughout (*I*^2^ ranging from 72% to 79%), reflecting persistent between-study variability regardless of individual study exclusion.

### Certainty of evidence

The certainty of evidence was generally limited across outcomes and study designs. For mortality, H-LOS, and AKI, randomized trials provided low-certainty evidence, primarily due to imprecision and between-study variability, whereas observational studies yielded very low-certainty evidence driven by risk of bias, heterogeneity, and wide confidence intervals. Detailed GRADE assessments are provided in the Supplementary Material.

## Discussion

This systematic review and Bayesian meta-analysis evaluated the impact of fluid accumulation—defined by either liberal fluid strategies or positive fluid balance—on clinical outcomes in adult patients undergoing cardiac surgery. Our findings indicate that while some outcomes appear consistently associated with fluid accumulation, others remain uncertain or inconclusive.

Both statistical frameworks suggest a likely harmful effect of liberal fluid strategies on all-cause mortality. The frequentist model demonstrated a statistically significant increase in mortality, and Bayesian modeling supported this with high posterior probabilities of benefit favoring restriction across all priors. Although the heterogeneity was substantial, the alignment between both methods reinforces the credibility of this finding. For AKI, Bayesian analysis also indicated a high probability of benefit from fluid restriction. However, the lack of significance in the frequentist analysis and the presence of high heterogeneity call for cautious interpretation. These estimates offer valuable insight but should not be regarded as definitive in isolation.

The H–LOS was reduced in the frequentist analysis and showed a moderately high probability of benefit in Bayesian analysis. However, the substantial heterogeneity and sensitivity to prior assumptions limit the strength of this conclusion. Subgroup analysis suggested more pronounced effects in observational studies compared with RCTs, although the variability remained high.

No consistent association was observed for POAF, ICU–LOS, mechanical ventilation duration, or ICU readmissions. Both statistical approaches produced neutral or inconclusive findings, suggesting either no true effect or insufficient power and consistency in available data.

Our dataset therefore brings together two complementary perspectives on perioperative fluid exposure: trials that tested protocol-based fluid strategies and observational studies describing achieved states of fluid accumulation (e.g., positive fluid balance, weight gain, cumulative overload). These constructs are not identical—strategy trials aim to prevent or limit harmful accumulation states, whereas observational definitions capture the degree of exposure that occurred under routine care—but both point towards the same clinical problem of excessive fluid burden. In our analyses, protocol-based trials were treated as a distinct exposure category in subgroup and sensitivity analyses, and excluding these trials did not materially change the overall direction of the association. This is consistent with broader evidence that fluid accumulation is associated with worse outcomes in surgical and non-surgical populations [2, 37–41] and with plausible pathophysiological mechanisms, including microcirculatory impairment due to increased diffusion distance and hemodilution [42], disruption of the endothelial glycocalyx with increased vascular permeability and tissue edema [42, 43], and elevated venous pressure leading to reduced organ perfusion pressure and organ dysfunction—particularly in encapsulated organs such as the kidneys [44, 45]. Higher fluid volumes also promote hemodilution, potentially lowering hemoglobin concentrations, influencing transfusion thresholds, and further modulating oxygen delivery and organ perfusion; however, transfusion-related outcomes were rarely reported in relation to fluid exposure, and only one included trial explicitly evaluated this association. Cardiac surgery patients are especially vulnerable to these mechanisms, and recent ERAS/STS expert consensus [46] recommends goal-directed fluid therapy (GDT) to reduce the risk of fluid overload and improve postoperative recovery [47, 48].

This review has several strengths. To our knowledge, this is the first meta-analysis to evaluate the impact of fluid accumulation on the clinical outcomes of patients undergoing cardiac surgery. While previous meta-analyses have investigated the effects of GDT or the type of fluid used in this population [48, 49], none have specifically addressed the direct impact of fluid accumulation. Additionally, the inclusion of over 15,000 patients across 18 studies enhances the statistical power and generalizability of our findings. By synthesizing evidence through both frequentist and Bayesian frameworks, we aimed to provide a more clinically grounded understanding of how fluid burden itself—rather than fluid delivery strategy alone—impacts patient outcomes.

Another strength is the use of a dual analytical framework combining both frequentist and Bayesian approaches. This enabled a probabilistic interpretation of the results, providing additional insight, especially in the presence of substantial heterogeneity and borderline significance in frequentist analysis. Furthermore, this review employed a comprehensive and systematic search strategy across multiple databases, supplemented by backward and forward citation tracking to ensure that all relevant studies were captured. Quality assessment was meticulously conducted using validated tools (ROBINS-I for observational studies and RoB 2 for randomized trials), and sensitivity analyses—including leave-one-out testing—confirmed that no single study disproportionately influenced the results.

When interpreted through the GRADE framework, the certainty supporting the associations identified in this review is limited. For mortality, only one randomized trial contributed data, yielding low-certainty evidence due to substantial imprecision, while the observational body of evidence was rated very low certainty because of serious risk of bias, inconsistency, and imprecision. For AKI and hospital length of stay, the randomized evidence also provided low certainty, and observational studies again contributed very low-certainty evidence, largely driven by heterogeneity and residual confounding. However, despite these limitations, the consistency in the direction of effect, the biological plausibility grounded in fluid physiology, and the high posterior probabilities observed in Bayesian analyses collectively suggest that fluid accumulation is unlikely to be a benign phenomenon. Rather than detracting from the findings, the GRADE evaluation highlights a critical evidence gap and reinforces the need for larger, rigorously designed randomized trials capable of testing whether restrictive or physiologically guided strategies can meaningfully improve postoperative outcomes.

This study has several limitations. First, there is substantial conceptual heterogeneity in how fluid accumulation was defined across trials, ranging from intraoperative balance to early postoperative balance, cumulative or weight-based overload, and protocol-based fluid strategies. In exploratory subgroup analyses based on these categories, the association between greater fluid accumulation or more liberal strategies and adverse outcomes was directionally consistent, but estimates were imprecise and heterogeneity often persisted. Thus, our findings support the plausibility that excessive perioperative fluid accumulation is harmful, but they do not establish a single universal threshold applicable to all settings and definitions. As highlighted by Malbrain and colleagues [50], “fluid accumulation” may even occur in the presence of euvolemia or hypovolemia, further underscoring the complexity of this construct.

Second, the number of RCTs was limited, and most of the evidence came from observational studies, which are inherently prone to confounding and selection bias despite statistical adjustment.

Third, the high statistical heterogeneity observed for outcomes that reached significance in the frequentist analysis—such as all-cause mortality (*I*^2^ = 75%) and H–LOS (*I*^2^ = 98%)—may affect the generalizability and robustness of the pooled estimates. Although we explored sources of variability using definition-based subgroup analyses and sensitivity analyses, the small number of studies per subgroup and the lack of patient-level data precluded more granular approaches, such as meta-regression or multiple design-based subgroups. We also evaluated small-study effects using Egger’s test, although the limited number of studies reduced the power to reliably detect publication bias. Finally, while the Bayesian analyses help to frame the uncertainty related to between-study variability, the sensitivity of posterior distributions—particularly for H–LOS—reinforces the need for cautious interpretation of these findings.

## Conclusion

Fluid accumulation may be associated with adverse postoperative outcomes in patients undergoing cardiac surgery. However, the overall certainty of evidence is low and heterogeneity is substantial, so these results should be interpreted as hypothesis-generating rather than as a basis for firm recommendations about restrictive thresholds. Further large, high-quality randomized trials are needed to define safe fluid exposure limits and to clarify the role of individualized, physiologically guided fluid strategies in this high-risk population.

## Supplementary Information

Additional file 1: Table S1 – PRISMA Checklist. Table S2 – Detailed Search Strategy. Supplementary Figure 1. Supplementary Figure 2. Supplementary Figure 3. Supplementary Figure 4. Supplementary Figure 5. Supplementary Figure 6. Supplementary Figure 7. Supplementary Figure 8. Supplementary Figure 9. Supplementary Figure 10. GRADE Framework.

## Data Availability

This study is a systematic review and meta-analysis and does not involve the collection of new, unpublished primary data. All data underlying the findings were extracted from previously published studies, which are fully cited in the manuscript. The extracted datasets used for the analyses are available from the authors upon reasonable request. No restrictions apply to data sharing.

## Abbreviations

AKI: Acute kidney injury
CABG: Coronary artery bypass grafting
CI: Confidence interval
CrI: Credible Interval
GDT: Goal-Directed Therapy
H-LOS: Hospital Length of Stay
ICU: Intensive Care Unit
ICU-LOS: Intensive Care Unit Length of Stay
MCMC: Markov Chain Monte Carlo
MD: Mean Difference
OR: Odds Ratio
POAF: Postoperative Atrial Fibrillation
PRISMA: Preferred Reporting Items for Systematic Reviews and Meta-Analyses
PROSPERO: International Prospective Register of Systematic Reviews
RCT: Randomized Controlled Trial
ROBINS-I: Risk Of Bias In Non-randomized Studies of Interventions
RoB 2: Risk of Bias 2 (Cochrane tool for randomized trials)
SMD: Standardized Mean Difference
